# COVID-19 Disease, Women’s Predominant Non-Heparin Vaccine-Induced Thrombotic Thrombocytopenia and Kounis Syndrome: A Passepartout Cytokine Storm Interplay

**DOI:** 10.3390/biomedicines9080959

**Published:** 2021-08-05

**Authors:** Nicholas G. Kounis, Ioanna Koniari, Cesare de Gregorio, Stelios F. Assimakopoulos, Dimitrios Velissaris, Ming-Yow Hung, Virginia Mplani, Luca Saba, Aikaterini Brinia, Sophia N. Kouni, Christos Gogos, Mattia Giovannini, Elio Novembre, Vinu Arumugham, Darrell O. Ricke, George D. Soufras, Kenneth Nugent, Piero Sestili, Robert W. Malone

**Affiliations:** 1 Department of Cardiology, University of Patras Medical School, 26221 Patras, Greece; 2 Department of Cardiology, University Hospital of South Manchester NHS Foundation Trust, Manchester M23 9LT, UK; iokoniari@yahoo.gr; 3 Department of Clinical and Experimental Medicine, University of Messina Medical School, 98122 Messina, Italy; cesare.degregorio@unime.it; 4 Department of Internal Medicine, University of Patras Medical School, 26500 Patras, Greece; sassim@upatras.gr (S.F.A.); dimitrisvelissaris@yahoo.com (D.V.); 5 Division of Cardiology, Department of Internal Medicine, Shuang Ho Hospital, Taipei Medical University, New Taipei City 23561, Taiwan; myhung6@ms77.hinet.net; 6 Division of Cardiology, Department of Internal Medicine, School of Medicine, College of Medicine, Taipei Medical University, Taipei 110, Taiwan; 7 Taipei Heart Institute, Taipei Medical University, Taipei 110, Taiwan; 8 Intensive Care Unit, Patras University Hospital, 26500 Patras, Greece; virginiamplani@yahoo.gr; 9 Department of Radiology, Azienda Ospedaliero Universitaria di Cagliari, 09045 Cagliari, Italy; lucasaba@tiscali.it; 10 Allergy Practice, King George Square, 26221 Patras, Greece; kbrinia@yahoo.gr; 11 Speech Therapy Practice, Queen Olgas Square, 26221 Patras, Greece; snkouni@yahoo.gr; 12 Covid-19 Unit, Papageorgiou General Hospital, 56403 Thessaloniki, Greece; gogos-grivas@hotmail.com; 13 Allergy Unit, Department of Pediatrics, Meyer Children’s University Hospital, 50139 Florence, Italy; mattiag88@hotmail.it (M.G.); elio.novembre@unifi.it (E.N.); 14 Cisco Systems, Inc., San Jose, CA 95134, USA; vaccine.safety@aol.com; 15 Group 49 Biological and Chemical Technologies, Lincoln Laboratory, Massachusetts Institute of Technology, Lexington, MA 02421, USA; Darrell.Ricke@ll.mit.edu; 16 Department of Cardiology, Saint Andrews State General Hospital, 26221 Patras, Greece; gasouf@gmail.com; 17 Department of Internal Medicine, Texas Technical University Health Sciences Center, Lubbock, TX 79430, USA; kenneth.nugent@ttuhsc.edu; 18 Department of Biomolecular Sciences, Universita degli Studi di Urbino “Carlo Bo”, 61029 Urbino, Italy; piero.sestili@uniurb.it; 19 Vaccines and Biotechnology, Scottsville, VA 24590, USA; rwmalonemd@gmail.com

**Keywords:** anaphylaxis, COVID-19, cytokine storm, heparin, Kounis syndrome, thrombocytopenia, thrombosis

## Abstract

Coronavirus disease 2019 (COVID-19) and severe acute respiratory syndrome coronavirus 2 (SARS-CoV-2) constitute one of the deadliest pandemics in modern history demonstrating cardiovascular, gastrointestinal, hematologic, mucocutaneous, respiratory, neurological, renal and testicular manifestations and further complications. COVID-19-induced excessive immune response accompanied with uncontrolled release of cytokines culminating in cytokine storm seem to be the common pathogenetic mechanism of these complications. The aim of this narrative review is to elucidate the relation between anaphylaxis associated with profound hypotension or hypoxemia with pro-inflammatory cytokine release. COVID-19 relation with Kounis syndrome and post-COVID-19 vaccination correlation with heparin-induced thrombocytopenia with thrombosis (HITT), especially serious cerebral venous sinus thrombosis, were also reviewed. Methods: A current literature search in PubMed, Embase and Google databases was performed to reveal the pathophysiology, prevalence, clinical manifestation, correlation and treatment of COVID-19, anaphylaxis with profuse hypotension, Kounis acute coronary syndrome and thrombotic events post vaccination. Results: The same key immunological pathophysiology mechanisms and cells seem to underlie COVID-19 cardiovascular complications and the anaphylaxis-associated Kounis syndrome. The myocardial injury in patients with COVID-19 has been attributed to coronary spasm, plaque rupture and microthrombi formation, hypoxic injury or cytokine storm disposing the same pathophysiology with the three clinical variants of Kounis syndrome. COVID-19-interrelated vaccine excipients as polysorbate, polyethelene glycol (PEG) and trometamol constitute potential allergenic substances. Conclusion: Better acknowledgement of the pathophysiological mechanisms, clinical similarities, multiorgan complications of COVID-19 or other viral infections as dengue and human immunodeficiency viruses along with the action of inflammatory cells inducing the Kounis syndrome could identify better immunological approaches for prevention, treatment of the COVID-19 pandemic as well as post-COVID-19 vaccine adverse reactions.

## 1. Introduction

The latest threat to global health is the ongoing outbreak of the respiratory disease caused by SARS-CoV-2, named COVID-19, firstly recognized in December 2019 in the city of Wuhan, in Hubei province, China [1]. COVID-19, caused by SARS-CoV-2, constitutes one of the deadliest pandemics inmodern history. In a modern overpopulated world of almost 8 billion people, characterized by dramatic changes in environmental conditions, in conjunction with rapid development of intercontinental transportation and inadequate global public health mechanisms, viral diseases with significant infectivity may turn into global health threats. Whereas, the cardiovascular, gastrointestinal, hematologic, mucocutaneous, respiratory, neurological, renal and testicular manifestations, and further complications that concern the whole human pathology, can provide also the substrate for elucidation of the disease pathophysiology. The COVID-19 pandemic has been spreading worldwide, including to all of Europe and the United States. Careful identification of any similarities regarding clinical manifestation and subsequent multiorgan complications could provide a better acknowledgement of the underlying pathophysiology and trigger mechanisms, elucidating potential prevention and therapeutic strategies.

## 2. Methods

A literature search was conducted on the PubMed, MedLine, Embase databases and Google and updated on 28 February 2021 with the keywords “COVID-19”, “Kounis syndrome”, “cytokine storm”, “SARS-CoV-2”, “SARS-CoV”, “MERS”, “allergy”, “anaphylaxis”, “coronaviruses”, “mast cells”.

Bibliographic search was also undertaken. Articles in this review needed to be published up to end of June 2021, available as full text in English, categorized as original research, reviews, meta-analyses or letters to the editor. Database screening was closed on 28 June 2021.

Titles and abstracts were reviewed to verify these criteria. The articles were read in full if all inclusion requirements were present or if this remained unclear.

Searching references included in the manuscripts was an additional literature. The abstracts were scanned to assess their appropriateness to be included in this narrative review.

## 3. Results

### 3.1. Virology and Origin

Coronaviruses are enveloped, positive single-stranded RNA viruses (+)RNA) with a genome of 27–32 kb. COVID-19 belongs to the beta-coronavirus genera, while evolutionary analyses have demonstrated bats and rodents as gene sources [2]. Regarding its origin, theories for laboratory construction have been widely spread through social media, but genetic data are not suggestive of this scenario. The receptor-binding domain in the spike protein is the most variable part of the coronavirus genome. Genetic manipulations in laboratories have been performed on available viral reverse-genetic systems, allowing researchers to introduce scheduled mutations. However, genetic data clearly reveal that COVID-19 is not derived from any previously used virus backbone, supporting the evidence that COVID-19 is a novel coronavirus, originated from natural selection, either in an animal host pre or post zoonotic transfer [3].

Infection is initiated with virus attachment to its cellular receptor on the host cell surface. COVID-19 spike protein (S-protein) binds with the angiotensin-converting enzyme 2 (ACE2) receptor on the epithelial cells’ membrane. COVID-19 transmission via the respiratory system could be facilitated by the abundant ACE2 expression by human respiratory epithelium [4]. Given that ACE2 is also expressed in vascular endothelium, cardiovascular, gastrointestinal, brain, and renal tissue as well as testicular epithelium, these organs are rendered potential targets for infection [5]. ACE-2 metabolizes angiotensin II to the vasodilatory and anti-inflammatory peptide angiotensin. SARS-CoV-2 interrupts the metabolism of angiotensin II, which further results in increased angiotensin II that can cause vasoconstriction, endothelial and platelet activation, and proinflammatory cytokine release.

Although patients infected with COVID-19 can be asymptomatic or present mild symptoms, some patients may develop moderate or severe complications, such as acute respiratory distress syndrome (ARDS), acute cardiac injury, acute coronary syndromes, coagulopathy with thromboses, renal and hepatic dysfunction, accompanied by increased risk of mortality [6]. A common pathogenetic mechanism for these complications seems to be COVID-19-induced pro-inflammatory state, the so-called cytokine storm syndrome (CSS) [6].

### 3.2. SARS-CoV-2 and Other Human Coronaviruses

Up to now, seven coronaviruses have been identified that can cause human disease. Coronaviruses hCoV-HKU1, hCoV-OC43, hCoV-NL63 andhCoV-229E principally cause asymptomatic or mild respiratory and gastrointestinal infections, accounting for approximately 5–30% of common colds [7]. However, in the last two decades, three human coronaviruses resulted in outbreaks that raised considerable global health consternation. First, the severe acute respiratory syndrome coronavirus (SARS-CoV) was recognized in 2002 as the cause of the severe acute respiratory syndrome (SARS) infecting over 8000 people the next year (2003) and causing 774 deaths with a case fatality ratio of 9.5%. Nine years later, a new coronavirus appeared in the Middle East, causing a highly lethal respiratory disease (34% case fatality rate), and this virus was named MERS-CoV [7]. Finally, since December 2019, the new human coronavirus SARS-CoV-2 has rapidly spread from Wuhan, China, to over 223 countries and regions in the world, causing a global pandemic affecting 114 million people, with over 2.5 million deaths by 1 March 2021 and a case fatality ratio ranging from 1 to 3.5% in most countries [8]. The higher reproduction number (R0) estimated as 3.1 (0.58 for SARS-CoV and 0.69 for MERS) has contributed to this global distribution [9] (Table 1). It is anticipated that these rates will change over time.

Regarding SARS-CoV-2 biological features, its genome consists of 6 common major ORFs for coronaviruses, with SARS-CoV-2 sequence disposing a 70% similarity to SARS-CoV and nearly 40% to that of MERS-CoV.ORF1a and the sequence of gene spike coding protein-S, the pivotal protein that interacts with the target cells, constitute the main differences among these three viruses [10]. SARS-CoV and SARS-CoV-2 S proteins are both attractive to ACE2 by electrostatic forces; however, mutations between SARS-CoV S protein and SARS-CoV-2 S protein lead to electric field line different densities, which results in a stronger interaction between SARS-CoV-2 and ACE2 [11].

These highly pathogenic human coronaviruses have evolutionarily acquired the ability to encode numerous proteins that allow them to escape the immune response, but also to overactivate inflammatory and immune cells, inducing a cytokine storm [7]. Severe systemic complications, such as coagulopathy with thromboses, acute cardiac injury, brain, liver injury and multiple-organ dysfunction have been associated with the cytokine storm syndrome, leading to increased risk of mortality. The characteristics and clinical manifestations of this cytokine storm syndrome have been described with SARS-CoV-2 owing to the abundance of human cases observed.

### 3.3. The Cytokine Storm

Cytokine storm is a complex and excessive immune response accompanied by excessive or uncontrolled release of pro-inflammatory cytokines that can be triggered by a variety of external stimuli such as severe viral infection as influenza [12], certain drugs [13] and cancer immunotherapeutic agents [14].

Cytokine storm has also been described as an unfortunate consequence and adverse effect of some monoclonal antibody medications as well as adoptiveT-cell therapies [14,15,16]. In addition, severe anaphylactic reactions with profuse hypotension or hypoxemia can be also associated with the release of pro-inflammatory cytokines [17].

The term “cytokine storm” was first used in 1993, in a case of a graft-versus-host disease [18]. Its association with infectious diseases started in early 2000 in several reports related to hemophagocytic lymphohistiocytosis/macrophage activation syndrome (HLH/MAS) [19], group A streptococcus [20], influenza virus [21], variola virus infections [22], and SARS-CoV-2 [23]. In 2005, it was applied in the context of the avian H5N1 influenza virus infection [24]. During the H1N1 influenza pandemic, the cytokine storm was also considered as the cause of death among several victims [25].

In cancer immunotherapy, with chimeric antigen receptor T-cells (CAR-T) treatment, autoimmune toxicity manifesting as CSS results from an antigen-specific attack on host tissues when the targeted tumor-associated antigen is expressed as well on non malignant tissues [26].

Another condition, the so-called cytokine release syndrome (CRS), is increasingly recognized, and it is caused by high-level immune activation, constituting anon-antigen-specific toxicity. Such high immune activation is necessary to mediate clinical beneficial effects via modern immunotherapies [14].

In severe anaphylaxis with either hypotension or hypoxemia, a number of cytokines are released and elevated in blood serum during the acute phase, correlating with the presence of hypotension [27]. During the allergic respiratory reactions, mediators may be largely confined to the airways’ tissue, without concomitant increase in blood serum levels. Alternatively, other mediators such as anaphylatoxins may play a significant role during respiratory reactions [28].

The cytokine storm is considered to be one of the major causes of ARDS, usually associated with multiple-organ failure [29], playing an important role inthe process of disease aggravation [30,31].

In critically ill patients, suffering from COVID-19 infection, the cytokine storm can deteriorate even more their clinical condition; therefore, its effective detection and suppression is of paramount importance for a positive outcome.

### 3.4. The Cytokine “Network”

The cytokine storm and the consecutive dangerous complications arise from the combined effects of several immune-active molecules (Table 2). The different types of cytokines associated with the cytokine storm include [13,32,33]:

- Interleukins (ILs), so named because their fundamental function appears to be the communication between (inter-) various populations of white blood cells (leucocytes-leukin) and further regulation of the immune cell differentiation and activation. They are produced by almost all stromal cells, mast cells, B lymphocytes, T lymphocytes, macrophages, monocytes, dendritic cells, and other non-lymphocytic cells, such as fibroblasts, endothelial cells, keratinocytes, glomerular mesangial cells and tumor cells [34]. They induce an increase of the acute phase signaling, activate epithelial cells, and mediate the transportation of immune cells to the infection site, triggering also the production of secondary cytokines [35]. Interleukins comprise the largest group of cytokines.
- Chemokines are small secreted proteins that can bind to one or more of 21 G-protein-coupled receptors. Chemokines serve as chemoattractants to recruit inflammatory cells from endothelium and epithelium into the inflammation site [36], especially those of the immune system, contributing to innate and adaptive immune function and development. The role of one specific chemokine, CXCL10 (previously referred to as interferon-γ inducible protein of 10 kDa, or IP-10), has been highlighted in ARDS and coronary syndromes [37]. Chemokines are emerging as the second largest family of cytokines.
- Interferons (IFNs), so named because they interfere with virus replication and play the central role in establishing innate immunity to viruses and other microbial pathogens [38,39].
- Tumor necrosis factors (TNFs), called by their ability to cause a hemorrhagic tumor necrosis post injection into experimental animals, are involved in the pathogenesis of septic shock. They are regarded, today, as the central cytokines in acute viral diseases, including influenza virus, dengue virus, and Ebola virus diseases. Immune cells can release TNFs in the acute inflammation and infection phase, while they have been correlated with several chronic inflammatory and autoimmune diseases [40,41].
- Colony-stimulating factors (CSFs) play an important role supporting the growth and differentiation of various bone marrow elements. They are proteinic components of a cascade that induces cytokine production by macrophages at sites of inflammation, further perpetuating the inflammatory reaction [42]. They divide into different subtypes based on their action as: granulocyte colony-stimulating factor (G-CSF), macrophage colony-stimulating factor (M-CSF), and granulocyte–macrophage colony-stimulating factor (GM-CSF). Ongoing trials will help to inform whether blunting the inflammatory signaling provided by the G-CSF axis in COVID-19 is beneficial [43].

### 3.5. Cytokine Storm Clinical Manifestations

The cytokine storm clinically manifests during activation and further release of inflammatory cytokines by large numbers of lymphocytes (B lymphocytes, T lymphocytes, and/or natural killer cells) and/or myeloid cells (macrophages, mast cells, monocytes, eosinophils, dendritic cells, or even endothelial cells lining blood vessels) [44]. Pleiotropism characterizes each individual cytokine that can exert multiple functions depending upon the cell that produces it and the target cell(s) upon which it acts. The severity of cytokine storm and the symptom onset may vary according to the inducing cause and the magnitude of immune cell activation. The clinical symptomatology of the cytokine storm includes a variety of symptoms and signs (Table 3) that range from mild, flu-like symptoms to severe life-threatening manifestations, depending on the severity of inflammatory response. Arthralgia, fatigue, fever, headache, muscular pains and rash constitute mild symptoms, but in severe cases, hypotension, hypoxemia and fever can further lead to severe and uncontrolled systemic inflammatory response accompanied by vasodepressor-circulatory shock, and vascular leakage, as in anaphylactic shock [45]. Furthermore, thrombotic vascular events, disseminated intravascular coagulation, and multiorgan system failure may be induced.

Respiratory complications can often occur in patients with cytokine storm. Cough and tachypnea may be prevalent in mild cases that can also progress to ARDS characterized by dyspnea, hypoxemia, and evidence of bilateral opacities on chest X-ray [44]. Mechanical ventilation might also be required in ARDS due to airway protection inability secondary to neurotoxicity rather than actual respiratory distress [46].

Neurologic symptoms and signs are also encountered during cytokine storm [47] and range from mild confusion, hallucinations, headaches, word-finding difficulty, to aphasia, cranial nerve palsies, hemiparesis, seizures, and somnolence.

Cardiovascular complications of cytokine storm in general, and in viral infections in particular, may include arrhythmias, myocarditis, myocardial ischemia, pericarditis, and type 1 (atherosclerotic plaque rupture) and type 2 (secondary to an ischemic imbalance) myocardial infarction and heart failure [48].

### 3.6. COVID-19, Kawasaki Disease and Chimeric Antigen Receptor T-Cell (CAR-T) Therapy-Associated Cytokine Storm

Recent reports have demonstrated cases of children suffering from COVID-19 with unusual febrile illnesses, acute abdominal conditions, toxic shock syndrome, encephalopathy, elevated inflammatory markers, and multisystem involvement that resemble the features of Kawasaki’s disease (KD). Kawasaki disease is an acute inflammatory disease characterized by medium-sized vasculitis with predilection for coronary arteries, predominantly affecting children. Coronary artery aneurysms from KD may manifest in adult life, accounting for 5% of acute coronary syndromes in respective individuals. Kawasaki syndrome is associated with a variety of viruses [49] such as adenovirus, Coxsackie B3 virus, dengue virus, Epstein–Barr virus, herpes virus, human bocavirus, human coronavirus, human immunodeficiency virus, influenza virus, measles, para-influenza type 3, parvovirus B19, rotavirus, Varicella-Zoster Virus and even 2009 H1N1 virus.

Bacteria and viruses have been sporadically isolated from KD patients and their proteins acting as superantigens proposed as possible triggers of a dysregulated immune response.The presence of tissue intracytoplasmic inclusion bodies and RNA virus-like inclusion bodies in KD patients supports the hypothesis that RNA viruses are closely involved in the disease etiopathogenesis [50].

In many pediatric COVID-19 patients, a new nosological entity of a multisystem cytokine storm temporally associated with SARS-CoV-2 has been documented and recognized as a multisystem inflammatory syndrome in children (MIS-C). This typically occurs 2–6 weeks after acute SARS-CoV-2 infection. Symptoms include persistent fever, gastrointestinal issues, multiorgan inflammation, low blood pressure, high inflammatory markers, and a variety of other symptoms, including respiratory. Hospital admission is mandatory and approximately 60% of patients are admitted to intensive care unit and approximately 2% die.

The pathogenic mechanisms are not well understood, but the delay between infection/exposure and onset suggests inappropriate innate immune responses elicited by infection, sometimes leading to a “cytokine storm syndrome” (CSS) [51]. A condition resembling MIS-C, called MIS-A, has also been reported, but more rarely, in adults [52]. It is not clear, however, that this condition pathogenesis is identical to MIS-C. MIS-C shares several features with Kawasaki disease, but differs from the cytokine storm of severe acute COVID-19 [53]. It also differs from this condition with respect to T-cell subsets, interleukin (IL)-17A, and biomarkers associated with arterial damage. Additionally, MIS-C occurs in older children (median age 9–10 years in largest series of African origin, while KD predominantly occurs in children ≤5 years of age of Asian descent. MIS-C is associated with more severe lymphopenia, higher levels of the inflammatory markers C-reactive protein and ferritin, and lower platelet counts compared to Kawasaki disease. Significantly elevated inflammatory cytokines are present in MIS-C including IL-6, IL-17A, and IL-18, and elevated chemokines as CCL3, 4, 20, and 28, and CXCL10. There are also reductions of subsets of natural killer and T lymphocytes. Multiple autoantibodies against self-antigens, such as La, a lupus autoantigen, and Jo-1, seen in idiopathic inflammatory myopathies, might be present. Thus, MIS-C appears to be both autoinflammatory and autoimmune in nature. Interestingly, treatment with IL-6R antibody or IV immunoglobulin has led to disease resolution [54]. Other treatments commonly used include IL-1RA or a high dose of steroids.

Another condition associated with cytokine storm that is related to the COVD-19 pandemic is the Chimeric antigen receptor T-cell (CAR-T) therapy used to patients with relapsed/refractory hematologic malignancies. Whereas such a therapy is a successful therapeutic strategy for improving clinical outcomes, unexpected complications and challenges amid the COVID-19 pandemic may be encountered. Indeed, patients with hematologic malignancies could be severely affected by SARS-CoV-2 infection mainly because of immunosuppression. The most common adverse effect of CAR-T therapy is CSS, which is triggered by the binding of chimeric antigen receptors with their target antigens [55]. Generally, CSS is reversible and only requires supportive therapy, but in some extreme cases, it can be life-threatening. CSS is characterized by the activation of epithelial cells, which may result in pulmonary edema, eventually leading to acute respiratory failure in severe cases. Previous studies have explored the correlation between cytokine profiles and the severity of CSS after CAR T-cell infusion. Early elevated serum levels of multiple cytokines, including interleukins IL-6, IL-8, and IL-10, monocyte chemoattractant protein 1 (MCP-1), macrophage inflammatory protein-1β (MIP-1β), and interferon-g (IFN-g), can be associated with the development of severe CRS [56]. Patients who have completed CAR-T treatment and suffered as well from confirmed COVID-19 infection, should be treated based on well-acknowledged guidelines [57] and local medical guidelines for the diagnosis and treatment of COVID-19. On the other hand, high-dose steroids should be avoided, as this could disrupt CAR-T cell persistence and may affect the outcomes of CAR-T therapy. In addition, tocilizumab or artificial liver treatment is recommended for severe COVID-19 cases with elevated cytokine levels. Indeed, the artificial liver is recommended to inhibit pro-inflammatory cytokine cascade. All medical staff should use preventive measures and rules during ward rounds to avoid cross-infection, whereas the use of disposable caps, surgical masks, and other garments is imperative. Proper hand washing and disinfection with alcohol or hydrogen peroxide should also be performed by both staff members and patients [57]. A comparison of characteristics of SARS-CoV-2 CSS, CAR-T cell therapy-associated CRS and HLH/MAS is shown in Table 4.

### 3.7. Cytokine Storm in COVID-19, Cytokine Surge in Anaphylaxis

The immediate-type of generalized hypersensitivity reaction that affects multiple organ systems and manifests, at its most severe form, by bronchospasm, upper airway obstruction, hypoxemia, hypotension, collapseand cardiovascular complications including Kounis syndrome [62] is anaphylaxis (an-aphylaxis means ‘no-prophylaxis’, ‘opposite protection’ or ‘against protection’, whereas prophylaxis in Greek means ‘protection’).

Since the era of histamine thought to be the principal mediator of anaphylaxis, a range of other mediators, similar to those of cytokine storm in COVID-19 patients, have been implicated in human anaphylaxis, in vitro cell stimulation studies, and in animal models [63]. Mast cells and other inflammatory cells can release anaphylaxis-implicated mediators resembling cytokine storm molecules. These mediators include beyond histamine, mast cell tryptase, chymase, and cathepsin-D, newly synthesized cytokines/chemokines such as tumor necrosis factor-a (TNF-a), IL-1, IL-4, IL-5, IL-6, IL-8, IL-9, IL-13, complement breakdown products such as the anaphylatoxins C3a, C4a, and C5a, contact system activation products including bradykinin, lipid-derived mediators (platelet activating factor, prostaglandin D2, leukotriene LT B4, cysteinyl leukotrienes LTC4, LTD4, and LTE4), and a variety of pro- or anti-inflammatory products stemmed from eosinophil activation [64].

**Figure 1:**
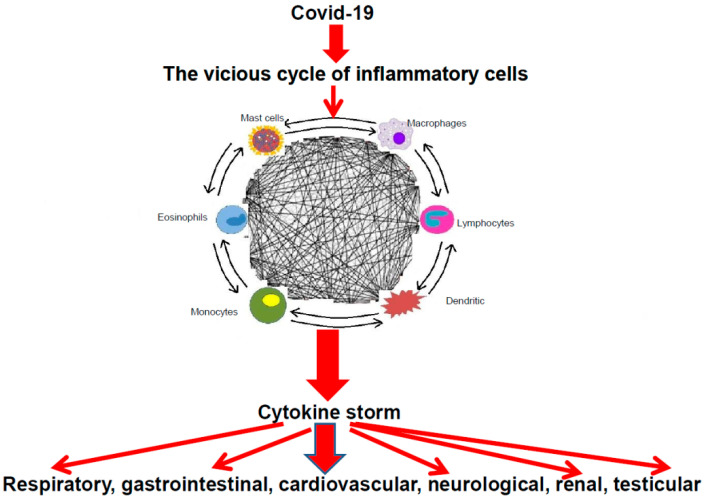
The cascade of COVID-19 and the vicious cycle of inflammatory cells leading to cytokine storm with their clinical consequences.

Indeed, mast cells and other inflammatory cells can activate each other via multidirectional signals like a “ball of thread”, participating in an inflammatory vicious cycle (Figure 1). For instance, mast cells can induce macrophage activation [65] and enhance T-cell activation [66], whereas mast cells might be activated by inducible macrophage protein 1a [67]. In addition, CD169-macrophages can induce CD8 T-cell activation [68], which, in turn, may mediate mast-cell activation and proliferation [69] and regulate macrophage activity [70].

Monocyte chemotactic protein 3 is a most effective basophil-and eosinophil-activating chemokine [71]. Dendritic cells may initiate autoimmune responses and stimulate T cells with resultant macrophage activation [72].

Interactions between lymphocytes, monocytes, macrophages, and mast cells increase vascular permeability, via release of TNF, IL-1β, IL-6, CXCL8 (IL-8), macrophage migration inhibitory factor, CCL2 (also known as monocyte chemoattractant protein-1, MCP-1), high mobility group box-1 and matrix metalloproteinases [73]. The latter can promote plaque disruption or rupture, leading to myocardial infarction via activation of their zymogen forms interstitial collagenase, gelatinase, and stromelysin [74].

### 3.8. Viral Infections and Mast Cells

Viral infections seem to interplay mast cell activation cascade [75], as mast cells participate in certain viruses host responses, but their precise role remains to be elucidated [76]. Moreover, mast cells can be insulted by some viruses as dengue virus and human immunodeficiency virus (HIV) [77,78]. There is evidence that human mast cells can constitute a persistent viral infection reservoir as mast cell exposure to TLR2 (toll-like receptors), −4, or −9 ligands can trigger virus replication in latently infected cells. Moreover, virus co-receptor expression by mast cells might be influenced by IgE-FcεRI interactions, thus establishing their susceptibility to infection with CXCR4-tropic and R5X4-tropic variants [79].

Indeed, human and rodent mast cells express TLR3s that constitute a class of proteins expressed on sentinel cells such as macrophages and dendritic cells, which can recognize structurally conserved molecules derived from microbes [80]. These receptors can be activated by viral double-stranded RNA to release various chemokines and cytokines, including IFN-α and IFN-β [81].

Viral activation of mast cell-associated-cytokine production might also insult the airway tissue and the coronary arteries, culminating in ARDS and in immunology-associated Kounis syndrome [82].

It has been proposed that co-stimulation of human and rodent mast cell populations in vitro with FcεRI and certain TLRs can induce release of various pro-inflammatory mediators, suggesting another mechanism through which bacterial or viral infections might exacerbate atopic asthma and other IgE- and mast cell-associated disorders such as Kounis syndrome in vivo [76,81].

### 3.9. The Dengue and Human Immunodeficiency Viruses (HIV) Paradigm

Surprisingly, the same clinical manifestations as in COVID-19, namely fever, headache, bone pain, skin rash, shock syndrome with major features of high levels of pro-inflammatory cytokines, vascular leakage, thrombocytopenia, hypotensive shock, myocarditis, myocardial infarction and to a lesser extent hemorrhage [83], are encountered in other viral diseases including dengue fever-a mosquito-transmitted viral infection [84].

Dengue virus has been associated with immunological mechanisms such as antibody-dependent enhancement [85]. Indeed, opsonization of dengue particles can bound in immune complexes with non-neutralizing antibodies, leading to an increase in infection rate in FcR-bearing cells, such as mast cells, dendritic cells and monocytes and higher levels of viral replication [86].

In subjects infected with HIV, the IgEs, HIV-1 gp120 and Tat proteins were demonstrated to be increased [86]. These molecules can induce human mast cells migration, functional activation in vitro, and further release of IL-4 and IL-13 from human mast cells and basophils. Moreover, Tat protein also can upregulate the β-chemokine receptor CCR3, expressed on lung mast cells, that it also constitutes a co-receptor of HIV-1 infection [87].

Therefore, this cascade of viruses, coronaviruses, mast cells, other interacting inflammatory cells and platelets lead to releasing mediators acting on specific receptors. The final result is myocardial injury, coagulopathy, thrombosis, coronary complications and, in particular, the immunity-associated, induced by mast cell activation Kounis syndrome.

### 3.10. The Kounis Syndrome

Anaphylactic, allergic, hypersensitivity, or anaphylactoid reactions correlated with cardiovascular disorders were initially characterized as acute carditis, morphologic cardiac reactions, or rheumatic carditis of unknown pathophysiology and were attributed to serum pathology. The allergic angina syndrome, a coronary spasm leading to allergic acute myocardial infarction constituting an endothelial dysfunction or microvascular manifestation, was first introduced 30 years ago [88] and in 2005 was named Kounis syndrome [89].

The pathophysiology of this syndrome [90] includes inflammatory mediators released during an anaphylactic event from mast cell activation and degranulation in the context of other interacting cells, including lymphocytes, macrophages, eosinophils, monocytes, eosinophils and dendritic cells (Table 5). Although mast cells consist numerically as a minority in this inflammatory cascade, they influence decisively the inflammatory process. Platelets also participate in this cascade via activation of their corresponding receptors by thromboxane, histamine, and platelet-activating factor. Moreover, platelets express also high affinity IgE (FcεRI) and low affinity IgE (FcεRII/CD32) receptors that constitute potential targets for many antigens [91]. Potential trigger factors of the Kounis syndrome include drugs, metals, foods, environmental exposures, and clinical conditions.

### 3.11. COVID-19 and Myocardial Infarction

According to recent reports, ST segment-elevation myocardial infarction (STEMI), may represent the first clinical manifestation of COVID-19. Indeed, STEMI represented the first clinical manifestation of COVID-19 in 85.7% of patients who did not have a COVID-19 test result at the time of coronary angiography [89,90]. Therefore, further elucidation of the myocardial injury pathophysiology in patients with COVID-19 seems of paramount importance.

The Kounis syndrome induced by the release of inflammatory mediators through mast cell degranulation may manifest as coronary spasm due to endothelial dysfunction or microvascular angina in patients with normal or nearly normal coronary arteries constituting subtype MINOCA (myocardial infarction with non-obstructive coronary arteries), coronary thrombosis of type1 myocardial infarction and stent thrombosis [92]. Similarly, the myocardial injury in patients with COVID-19 has been attributed to coronary spasm, direct endothelial or vascular injury, plaque rupture and microthrombi, hypoxic injury or cytokine storm [93].

Recently, a series of patients suffering from COVID-19 revealed a higher-than-expected incidence of stent thrombosis [94], a fact that was attributed to the underlying hypercoagulable state [95]. Although thrombus aspiration was performed only in one patient, further histopathological study that may be valuable for identification of stent thrombosis cause has not taken place.

Moreover, none of the other patients were evaluated for IgEs, specific IgEs and other inflammatory mediators that could have confirmed the presence of any type of cytokine surge and Kounis syndrome [96]. Indeed, in a recent longitudinal analysis, severe COVID-19 was accompanied by an increase in effector cells including IgEs and eosinophils [97]. Furthermore, in current clinical practice, Kounis syndrome has appeared not only in COVID-19 cases [98,99], but also in cases following vaccine administration for prevention of COVID-19 disease [100,101].

Although stent thrombosis is a multifactorial complication, the implanted “antigenic complex” of nickel alloys, polymers, eluted drugs, in conjunction with concomitant oral antiplatelet drugs and environmental exposures can induce Kounis syndrome. Thus far, all clinical reports and pathologic findings in all patients and animal experimental studies point toward inflammation with thrombus infiltration of various interrelated and interacting inflammatory cells, including eosinophils, macrophages, T cells, and mast cells [102].

The histamine-2 receptor antagonist famotidine that blocks the histamine 2 receptor has significantly reduced the risk for death or intubation (adjusted hazard ratio (aHR) 0.42, 95% CI 0.21–0.85) and also the risk for death alone (aHR 0.30, 95% CI 0.11–0.80) in hospitalized patients with COVID-19 [103].

Indeed, famotidine pretreatment can prevent endothelial cell permeability attributed to histamine H2 activation [103]. It must be emphasized that famotidine and other antihistamines are drugs of choice for prevention and treatment of Kounis syndrome [104], as well as for prevention of myocardial ischemia/reperfusion injury induced by histamine H2 receptor activation [105] and further for peripheral vessel leakage prevention that is incriminated for the shock in anaphylaxis.

The value of corticosteroids is widely debated, but they have been widely used in syndromes closely related to COVID-19, including SARS-CoV, MERS, severe influenza, and community-acquired pneumonia. Corticosteroids are also the drugs of choice for the treatment of Kounis syndrome. According to RECOVERY trial, dexamethasone reduced the 28-day mortality in hospitalized patients with COVID-19 receiving invasive mechanical ventilation or oxygen at randomization to 29.3%, as compared with 41.4% in the usual care group [106].

Therefore, these similarities in pathophysiology, etiology, clinical cardiac manifestations and therapeutic approaches of the severe COVID-19 cardiac complications and anaphylaxis-associated Kounis syndrome might be proven as beneficial for future treatments based on the antigenic substrate of the latter.

The use, for example, of humanized monoclonal antibodies to clear free IgEs and downregulate FcεRI expression might represent a new era for the treatment or prevention of such events. This has been already demonstrated for Kounis syndrome via anti-Immunoglobulin E administration [96,107].

### 3.12. COVID-19 Vaccine-Induced Thrombotic Events

The COVID-19 pandemic necessitated the rapid development of various technologies’ vaccines. Some rare cases of anaphylaxis, immune thrombocytopenia and bleeding without thrombosis have been associated with the messenger RNA (mRNA)-based vaccines, namely the mRNA-1273 (Moderna) and BNT162b2 (Pfizer–BioNTech) [108]. However, rare delayed manifestations, mainly in women of less than sixty years of age resembling autoimmune heparin-induced thrombocytopenia with thrombosis (HITT) and especially serious cerebral venous sinus thrombosis(CVST), in the absence of heparin administration, have appeared 5–24 days after initial vaccination with both the adenoviral vector ChAdOx1 nCov-19 (AstraZeneca) and Ad26.COV2.S (Janssen/Johnson & Johnson) vaccines [109]. In the latter rare cases, almost every patient has been detected with high levels of antibodies to platelet factor 4 (PF4)-polyanion complexes.

**Figure 2:**
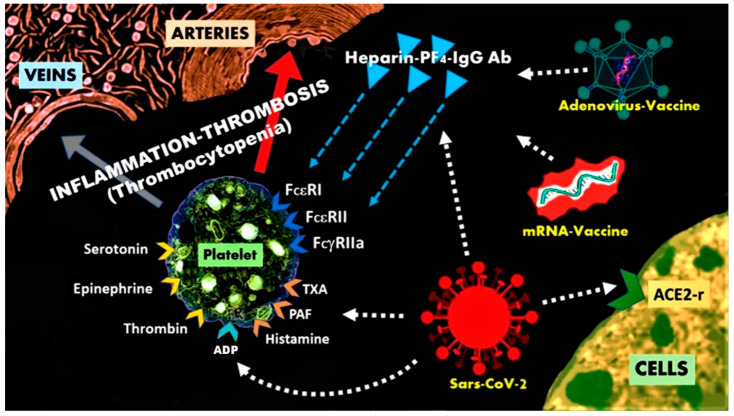
The heparin-PF4-IgG antibody complexes can activate platelets, via the FcεRI, FcεRII, FcγRII receptors and cause heparin-induced thrombocytopenia with thrombosis.

The arising questions, therefore, include: why these HIT-like thromboses are delayed and seem to occur in the absence of heparin administration with these 2 specific COVID-19 vaccines affecting only young middle-aged women? Is there any common pathway that connects all these 4 conditions? We believe that the following may provide an answer to these questions:

A. The prevalence of delayed adverse reactions and especially hypersensitivity reactions appearing days after vaccination is not new. Such reactions are antibody-independent, cell-mediated, stemming from over stimulation of T cells and monocytes/macrophages and cytokine’s release that further cause inflammation, cell death, and tissue damage [110]. They have been associated with vaccines containing anti-microbial agents and ingredients, such as thimerosal and aluminum [111] and withJapanese encephalitis and rabies vaccines [112]. A common type of delayed hypersensitivity reaction is the heparin hypersensitivity [113].
B. Heparin and related polymer heparin sulfate constitute natural glycosaminoglycans that have particular relevance to allergy and inflammation. The PF4 is a highly positive protein present in the a-granules of platelets that can quickly bind to either exogenous or endogenous heparin. Indeed, the platelet released PF4 binds to surface glycosaminoglycans on hematopoietic and vascular cells [114]. The pair PF4/heparin acts as an autoantigen and induces anti-PF4/heparin antibodies of IgG class. PF4-heparin-IgG antibody complexes have been detected in 3.1–4.4% of healthy subjects and can cause thrombosis via activation of the specific low-affinity IgG (FcγRIIa/CD32) receptors on the platelet surface. However, platelet surface disposes also high affinity IgE (FcεRI) and low affinity IgE (FcεRII/CD23) receptors, a fact that is not so well known to clinical practitioners [90]. Furthermore, receptors for histamine, platelet-activating factor, thromboxan, thrombin, adenosine diphosphate (ADP) can also exist in the platelet surface (Figure 2). Upon their activation, platelets secrete pro-inflammatory pro-thrombotic, adhesive, and chemotactic mediators that propagate, amplify and sustain the thrombotic process [115]. The platelet consumption, due to extensive thrombosis, leads to thrombocytopenia [116]. The 3-component PF4-heparin-IgG antibody complex can bind to FcγRII receptors on monocytes, inducing endothelial injury that leads to tissue factor release, thrombin production and diffusion. Thrombin production plays an important role in the pathogenesis of CVST and thrombosis in general [117]. The classical HIT is not complicated with CVST, despite constituting a highly pro-thrombotic condition. In COVID-19-vaccination-associated CVST, any form of heparin (i.e., unfractionated heparin, even for line flushes, or LMWH e.g., enoxaparin) and platelet transfusion should be avoided. Treatment with Argatroban, which is a direct thrombin inhibitor together with corticosteroids (that are used for Kounis syndrome) such as dexamethasone and immunoglobulin, are recommended [118]. All this cascade of actions, reactions, interactions, together with the released mediators and inflammatory cells, constitute the pathophysiologic basis of Kounis anaphylaxis-associated thrombotic syndrome [119]. It is reasonable, therefore, to anticipate that heparin is not always required to be given externally in order to cause HIT-like thrombosis and that HIT itself behaves as a new manifestation of Kounis syndrome [120,121].

C. Both adenoviral vector ChAdOx1 nCov-19(AstraZeneca) and Ad26.COV2.S (Janssen/Johnson & Johnson) vaccines contain excipients that could be potential antigens such as polysorbate, a synthetic nonionic surfactant, also known as Tween 80, which is a mixture of esters and etherates synthesized by oleic acid, ethylene oxide, sorbitan and isosorbide. Polysorbate 80 can induce systemic reactions including IgE immediate reactions as well as non-immunologic anaphylactoid reactions but also local reactions such as thrombophlebitis, pain, erythema [122]. Polysorbate can enhance membrane permeability, penetrate the blood–brain barrier and facilitate the passage of drugs from the blood compartment to the brain for therapeutic purposes, especially in oncology [123]. The release of cytokines from overstimulation of T cells, monocytes and macrophages can induce inflammatory response and delayed adverse reactions post polysorbate administration via the vaccine. Such polysorbate actions make one wonder whether or not the adenoviral vector vaccine-induced CVST is a simple coincidence. Indeed, as mentioned above, the classical HIT, which is not related to polysorbate, is not complicated with CVST.
D. Younger-aged women, who routinely use creams, ointments, lotions, and other cosmetics that contain polysorbate, could have been earlier sensitized to polysorbate and will likely develop adverse reactions when exposed to the same agent. Furthermore, polysorbate is also an ingredient in various dental materials and contact sensitization in dental patients has been proven [124]. Indeed, it is estimated that 1–5.4% of the population is already sensitized to cosmetics or cosmetic ingredients [124]. We suggest, therefore, that atopic subjects and patients with a previous allergic history should carefully look at SPC (Special Product Characteristics) of their used cosmetics for any containing polysorbate excipient, and also for any other ingredient cross-reacting with polysorbate such as polyethylene glycol product. The latter is contained in the messenger RNA (mRNA)-based vaccines mRNA-1273 (Moderna) and BNT162b2 (Pfizer–BioNTech), respectively. Additionally, the Moderna vaccine contains tromethamine, also known as trometamol, that is also used in cosmetic products as an emulsifier. Contact sensitization, allergy and anaphylaxis to this compound have been already reported [125]. Individuals who present late adverse reactions following the first dose of the above vaccines could proceed to skin testing in order to elucidate the cause of the reactions and in case of being positive to polysorbate 80 to avoid the second dose as subsequent exposure to the substance could even lead to an immediate type of reactions. Pharmaceutical companies should try to produce free allergenic vaccines. Free polysorbate oncology medications have been already in the market [122]. Alternatives to polysorbate and PEG excipients such as alkylsaccharides constitute promising agents because they reduced immunogenicity, improve stability, and suppress oxidative damage problems of polysorbates or other polyoxyethylene-based excipients [126].

## 4. Discussion

The Food and Drug Administration (FDA) has created a special emergency program forpossible therapies, the Coronavirus Treatment Acceleration Program, in an attempt to evaluate whether new treatments might be helpful and their further application to patient management as quickly as possible.

The main representatives of agents (Table 6) together with specific and non specific intravenous immune globulins, convalescent plasma and humanized monoclonal antibodies are available in the medical armamentarium. These medications seem to exert a positive impact, especially in severe cases with laboratory evidence of host hyper-inflammatory response and or development of the macrophage-activation syndrome.

In previous years, multiple viral and bacterial pathogens have been associated with atherosclerosis and experimental studies demonstrating an acceleration of atherogenesis following infection in animal models [127].

Recent studies have reported that COVID-19 infection enhances activation of the immune system similar to the macrophage-activation syndrome that may further unmask any potential subclinical cardiovascular disease promoting intra-plaque inflammation and subsequent pro-thrombotic activation [128].

Based on these patho-physiological mechanisms, it appears possible that asymptomatic subjects infected with COVID-19 are predisposed to an increased risk of conversion from asymptomatic, subclinical, atherosclerotic disease into an unstable state with vulnerable plaques prone to thrombosis [129].

Viral activated mast cells release early inflammatory chemical compounds, including histamine and proteases, while late activation may provoke theactivation of pro-inflammatory IL-1 family members including IL-1, IL-6, and IL-33. It has been proposed that inflammation by coronavirus may be inhibited by anti-inflammatory cytokines belonging to the IL-1 family members [130].

The role of Vitamin D has been shown to be protective and safe against acute respiratory infections, and dedicated studies about vitamin D levels inCOVID-19 have been proposed [131].

COVID-19 has been associated with ST segment-elevation myocardial infarction [115,132], but the course of the disease after their hospital discharge has not been clarified. Indeed, respiratory infections caused by influenza, para-influenza, rhinovirus, respiratory syncytial virus, adenovirus, or humanmeta-pneumovirus have been found as independent predictors of hospital readmission for myocardial infarction [133,134], while influenza epidemics are associated with a higher risk of out-of-hospital cardiac arrest [135]. Since pathophysiology and virulence mechanisms of coronavirus, and especially of COVID-19, are correlated with the function of their nonstructural and structural proteins [136], a similar effect could be expected and in patients with COVID-19infection. Implementation, therefore, of public health interventions may reduce the risk of out-of-hospital cardiac arrest during the current COVID-19 pandemic.

Whereas the current literature on COVID-19 is replete with anecdotal reports of therapeutic successes, small clinical trials, observational cohort studies, hypotheses, ideas and suggestions that claim efficacy and suspected success, it pays little attention to the effect of unrecognized confounders [137].

Indeed, several diverse medications with antiviral or immunomodulatory activity have been used in clinical practice, several others have been proposed, but large clinical trials that are necessary to prove their efficacy are lacking.

Therefore, physicians should be always bring in mind that “empty vessels make the most noise” expressed by the English author William Baldwin’s and Aristotle’s (384–322 B.C.) dictum: “Not many is the good, but in the good, the many” (Ouk en tō Pollo to af, but en to af to poly=OΥΚ ΕΝ ΤΩ ΠOΛΛΩ ΤO ΕΥ AΛΛA ΕΝ ΤΩ ΕΥ ΤO ΠOΛΥ)

## 5. Conclusions

Similarities between allergy/anaphylaxis-associated Kounis syndrome and COVID-19 immunology strongly suggest that the same key immune cells might be involved in COVID-19-induced anaphylaxis and the anaphylaxis-associated Kounis syndrome. It has been already emphasized that early immunological interventions targeting inflammatory markers predictive of adverse outcomes could be more beneficial than those targeting cytokine inhibition [119].

It seems that an individualized treatment approach, tailored to each patient, is required in COVID-19 cases; as severity of the disease, comorbidities, age, clinico-laboratory characteristics, indications for hyper-inflammatory response, cardiovascular and myocardial complications and host factors are important determinants for the disease progression. General and personal protective equipment for all people, and especially for the healthcare workers, is the final line of protection [138]. The incidence of severe adverse reactions and anaphylaxis is very low [139,140] but is highly encouraged to support vaccination against COVΙD-19 because vaccines are the only means to fight this pandemic. Further investigations of relevant risk identification and management approaches for COVID-19 consequences are required. There is much to learn from this pandemic.

## Figures and Tables

**Table 1:** Characteristics, similarities and differences among three coronaviruses [7,9].

	SARS-CoV-2	SARS-CoV	MERS-CoV
Mode of transmission	Droplet, aerosol, contact	Droplet, aerosol, contact	Droplet, aerosol, contact
Reproductive number (Ro)	3.1	0.58	0.69
Median incubation period	5 days	4 days	5.2 days
Cell entry receptors	ACE2	ACE2	Dipeptidyl peptidase 4
Disease course	diphasic	diphasic	Rapidly progressive
ARDS	18–30%	20%	20–30%
Imaging lung findings	Ground glass opacities, bilateral, multifocal, peripheral distribution	Ground glass opacities, unilateral focal and bilateral, multifocal, peripheral distribution	Ground glass opacities isolated unilateral and bilateral, multifocal, peripheral distribution
Lung pathology	Diffuse congestions with partly hemorrhagic necrosis	Edematous lungs with diffuse congestion and consolidation areas	Edematous lungs with consolidation
Acute kidney injury	3%	6.7%	41–50%
Fatality	1–3.5%	9.5%	34.4%
Antiviral treatments (reduce replication)	Remdesivir Favipiravir	Ribavirin Interferons Lopinavir and ribavirin	Ribavirin and interferon-a2a
Immunological treatments	Dexamethasone in SatO2 < 94% IL-1 inhibition IL-6 inhibition Janus kinase inhibition	Methylprednisolone controversial (interferons plus corticosteroids to reduce disease-associated impaired oxygen saturation)	(-)

**Table 2:** Cytokines constituting the cytokine storm.

Interleukins (ILs) IL-1 (IL-1β, IL-18, IL-33), IL-1α, IL-4, IL-5, IL-6, IL-8, IL-9, IL-10, IL-13
Chemokines (CXC, CC, C, CX3C) CXCR3, CXCL8, CXCL9, CXCL10, CXCL11, CCL2 (monocyte chemoattractant protein), CCL11 (eotaxin)
Interferons (IFNs) Type I ΙFNs (IFN-α, IFN-β) Type II IFN (IFN-γ) Type III IFNs IFN-λ1, λ2, λ3 (ΙL29) IL-28^α^ IL-28β
Tumor Necrosis Factors (TNFs)
Colony-Stimulating Factors (CSFs), Granulocyte colony-stimulating factor, Macrophage colony-stimulating factor

**Table 3:** Clinical signs, symptoms and laboratory findings associated with cytokine storm.

**Constitutional**	Anorexia, arthralgias, fever, fatigue, headache, myalgias, malaise, rigors
**Skin**	Edema, rash, rigor, urticaria
**Gastrointestinal**	Diarrhea, nausea, splenomegaly, vomiting
**Respiratory**	Hypoxemia, hypoxia, interstitial pulmonary edema, respiratory failure, tachypnea
**Cardiovascular**	Acute heart failure, arrhythmias, hypotension, increased cardiac output (early), potentially diminished cardiac output (late), myocarditis, stress cardiomyopathy, tachycardia, troponin elevation, QT prolongation, widened pulse pressure
**Blood and coagulation**	Bleeding, coagulopathy (increased PPT, reduced INR), cytopenias, disseminated intravascular coagulation, elevated D-dimers, febrile neutropenia, hypofibrinogenemia, hyperferritinemia
**Renal**	Acute kidney injury, azotemia, renal failure
**Hepatic**	Elevated liver enzymes, hepatomegaly, hyperbilirubinemia, liver failure
**Neurologic**	Altered gait, aphasia, confusion, delirium, difficulty in word finding, dysmetria, hallucinations, headache, mental status changes, paresis, seizures, tremor

**Table 4:** Characteristics of SARS-CoV-2 cytokine storm syndrome (CSS), CAR-T cell therapy-associated cytokine release syndrome (CRS) and hemophagocytic lymphohistiocytosis /macrophage activation syndrome (HLH/MAS) [58,59,60,61].

Characteristics	SARS-CoV-2- Associated CSS	CAR-T-Associated CRS	Hemophagocytic Lymphohistiocytosis/ Macrophage Activation Syndrome(HLH/MAS)
Cytokines	IL-2, IL-7, IL-10, G-SCF, IP10, MCP-1, MIP-1A, TNF-α	IFN-γ, IL-2, IL-2Ra, IL-6, sIL-6R, GM-CSF, IL-1, IL-10, IL-12, TNF-a, IFN-a, MCP-1, MIP-1A	TNF-α, IFNγ, IL-1, IL-6, IL-18
Pathologic cellular or cytokine driver	Impaired viral clearance, low levels of type I interferons, increased neutrophil extracellular traps (NETs)	Macrophages, CAR T cells, interleukin-6, interleukin-1β	CD8+ T cells, interferon-γ, interleukin-1β, myeloid-cell autoinflammation
Treatment	Glucocorticoids IL-1 inhibitor Il-6 inhibitor	IL-6 inhibitors TNF-α inhibitors Cyclophosphamide	Glucocorticoids IL-6 inhibitors TNF-α inhibitors
Differentiating laboratory features	Soluble IL-2 receptor-α low to normal Ferritin moderately to highly increased	Soluble IL-2 receptor-α very high Ferritin extremely high	Soluble IL-2 receptor-α very high Ferritin extremely high
Differentiating clinical features	Not specific but high rates of ARDS	Not specific	Not specific

**Table 5:** Mast cell: the “pleiotropic cell”and its inflammatory mediators participating in “Cytokine storm” able to induce the Kounis syndrome.

Preformed Mediators	Newly Synthesized Mediators
***Biogenic amines***	***Cytokines***
Histamine, Renin, angiotensin II, serotonin	Interleukins 1,2,3,4,5,6,9,10,13,16
	Interferon-γ
***Chemokines***	Macrophage activating factor
IL-8, MCP-1, MCP-3, MCP-4, RANTES (CCL5)	Tumor necrosis factor -a
***Enzymes***	***Growth factors***
Arylsulfatases, carboxypeptidase A, chymase, kinogenases, phospholipases, tryptase, cathepsin G	Granulocyte monocyte colony-stimulating factor Fibroblast growth factor Nerve growth factor, stem cell factor, VEGF
***Peptides***	***Arachidonic acid products***
Bradykinin, corticotropin-releasing hormone, endorphins, endothelin, somatostatin, substance B, vasoactive intestinal peptide, urocortin, vascular endothelial growth factor (VEGF)	Leucotrienes Platelet activating factor Prostaglandins Thromboxane
***Proteoglycanes***	
Chondroitin, heparine, hyaluronic acid	

**Table 6:** The variety of current COVID-19 treatments.

**Antivirals**	Darunavir, Favipiravir, Opinavir/Ritonavir, Oseltamivir, Remdesivir, Ribavirin, Umifenovir
**Anti-cytokine/anti-inflammatories**	Anakinra, canakinumab, eculizumab, sarilumab, tocilizumab
**Anti-coagulopathy drugs**	Heparin, low-molecular-weight heparins, dipyridamole
**Antiparasitics**	Ivermectin, nitazoxanide
**Colchicine**	
**Corticosteroids**	
**Cyclosporine**	
**Immunomodulatory/antivirals**	Azithromycin, Auranofin, Hydroxychloroquine/Chloroquine
**Interferons**	Type I IFNs (IFN-α and IFN-β)
**Janus kinase (JAK) inhibitors**	Baricitinib, ruxolitinib, tofacitinib
**Specific and non-specific intravenous immune globulins**	

## Data Availability

The study did not report new data.

## Abbreviations

aHR	Adjusted hazard ratio
ACE2	Angiotensin-converting enzyme 2
ADP	Adenosine diphosphate
ARDS	Acute respiratory distress syndrome
CAR-T	Chimeric antigen receptor T cells
CD8	Cluster of differentiation 8
CCL2	Chemokine (C-C motif) ligand 2
CCR3 C-C	chemokine receptor type 3
COVID-19	Coronavirus disease 2019
CRS	Cytokine release syndrome
CSS	Cytokine storm syndrome
CVST	Cerebral venous sinus thrombosis
CSFs	Colony-stimulating factors
GM-CSF	Granulocyte-macrophage colony-stimulating factor
G-CSF	Granulocyte colony-stimulating factor
FDA	Food and Drug Administration
FcγRIIa	Fragment crystallizable gamma region II alpha
FcεRI	Fragment crystallizable epsilon region I
FcεRII	Fragment crystallizable epsilon region II
HLH/MAS	Hemophagocytic lymphohistiocytosis/ macrophage activation syndrome
HIV	Human immunodeficiency virus
HIT	Heparin-induced thrombocytopenia
HITT	Heparin-induced thrombocytopenia with thrombosis
IFNs	Interferons
ILs	Interleukins
KD	Kawasaki disease
MAS	Macrophage activation syndrome
MERS	Middle East Respiratory Syndrome
MCP-1	Monocyte chemoattractant protein-1
M-CSF	Macrophage colony-stimulating factor
MINOCA	Myocardial infarction with non-obstructive coronary arteries
PAF	Platelet activating factor
PEG	Polyethylene glycol
RECOVERY	Randomized Evaluation of COVID-19 therapy
mRNA	Messenger ribonucleic acid
SARS-CoV-2	Severe acute respiratory syndrome coronavirus 2
SARS-COV	Severe acute respiratory syndrome coronavirus
SPC	Special product characteristics
STEMI ST	Segment-elevation myocardial infarction
TNFs	Tumor necrosis factors
TLR TXA	Toll-like receptor Thromboxane
